# Supplementary material for: End Sequence Analysis Toolkit (ESAT) expands the extractable information from single-cell RNA-seq data
Source: Genome Res. 2016 Oct;26(10):1397–410. doi: 10.1101/gr.207902.116 (PMC5052061; doi:10.1101/gr.207902.116)
Supplement: Supplemental Material [file supp_gr.207902.116_Supp_Materials.tar.gz › ESAT-esat_v0.1/doc/html/classumms_1_1core_1_1alignment_1_1_smith_waterman_alignment.html]

ESAT: umms.core.alignment.SmithWatermanAlignment Class Reference


ESAT


- Main Page
- Related Pages
- Packages
- Classes
- Files

- Class List
- Class Index
- Class Hierarchy
- Class Members

All Classes Namespaces Files Functions Variables Enumerator Pages

- umms
- core
- alignment
- SmithWatermanAlignment

Public Member Functions |
Static Public Member Functions |
Static Public Attributes |
List of all members

umms.core.alignment.SmithWatermanAlignment Class Reference

|  |  |
| --- | --- |
| Public Member Functions | |
|  | SmithWatermanAlignment (String seq) |
|  | |
|  | SmithWatermanAlignment (String seq, float matchScore, float mismatchScore, float gapOpenPenalty, float gapExtendPenalty) |
|  | |
| jaligner.Alignment | align (String seq) |
|  | |

|  |  |
| --- | --- |
| Static Public Member Functions | |
| static jaligner.Alignment | align (String seq1, String seq2, float matchScore, float mismatchScore, float gapOpenPenalty, float gapExtendPenalty) |
|  | |
| static String | getFullPrintableAlignment (jaligner.Alignment alignment) |
|  | |
| static int | ungappedMatchStartOnFirstSequence (String seq1, String seq2, float matchScore, float mismatchScore, float minPctIdentity) |
|  | |
| static int | matchStartOnFirstSequence (String seq1, String seq2, float matchScore, float mismatchScore, float gapOpenPenalty, float gapExtendPenalty, float minPctIdentity) |
|  | |
| static boolean | containsFullLengthUngappedMatch (String seq1, String seq2, int seq1start, int maxMismatches) |
|  | |
| static boolean | containsFullLengthUngappedMatch (String seq1, String seq2, int seq1start, float matchScore, float mismatchScore, int maxMismatches) |
|  | |
| static boolean | containsFullLengthUngappedMatch (String seq1, String seq2, int maxMismatches) |
|  | |
| static boolean | containsFullLengthUngappedMatch (String seq1, String seq2, float matchScore, float mismatchScore, int maxMismatches) |
|  | |

|  |  |
| --- | --- |
| Static Public Attributes | |
| static float | DEFAULT\_MATCH\_SCORE = 5 |
|  | |
| static float | DEFAULT\_MISMATCH\_SCORE = -4 |
|  | |
| static float | DEFAULT\_GAP\_OPEN\_PENALTY = 8 |
|  | |
| static float | DEFAULT\_GAP\_EXTEND\_PENALTY = 2 |
|  | |

## Constructor & Destructor Documentation

|  |  |  |  |  |  |
| --- | --- | --- | --- | --- | --- |
| umms.core.alignment.SmithWatermanAlignment.SmithWatermanAlignment | ( | String | *seq* | ) |  |

|  |  |  |  |
| --- | --- | --- | --- |
| umms.core.alignment.SmithWatermanAlignment.SmithWatermanAlignment | ( | String | *seq*, |
|  |  | float | *matchScore*, |
|  |  | float | *mismatchScore*, |
|  |  | float | *gapOpenPenalty*, |
|  |  | float | *gapExtendPenalty* |
|  | ) |  |  |

## Member Function Documentation

|  |  |  |  |  |  |
| --- | --- | --- | --- | --- | --- |
| jaligner.Alignment umms.core.alignment.SmithWatermanAlignment.align | ( | String | *seq* | ) |  |

Here is the caller graph for this function:

|  |  |  |  |
| --- | --- | --- | --- |
| static jaligner.Alignment umms.core.alignment.SmithWatermanAlignment.align | ( | String | *seq1*, |
|  |  | String | *seq2*, |
|  |  | float | *matchScore*, |
|  |  | float | *mismatchScore*, |
|  |  | float | *gapOpenPenalty*, |
|  |  | float | *gapExtendPenalty* |
|  | ) |  |  |

static

|  |  |  |  |
| --- | --- | --- | --- |
| static boolean umms.core.alignment.SmithWatermanAlignment.containsFullLengthUngappedMatch | ( | String | *seq1*, |
|  |  | String | *seq2*, |
|  |  | int | *seq1start*, |
|  |  | int | *maxMismatches* |
|  | ) |  |  |

static

Whether there is a full length ungapped match of sequence 2 to sequence 1, starting at specified position of sequence 1, allowing mismatches Uses default match and mismatch scores

Parameters
:   |  |  |
    | --- | --- |
    | seq1 | Sequence 1 |
    | seq2 | Sequence 2 |
    | seq1start | Required start position of match on sequence 1 |

Returns
:   Whether the best ungapped alignment is full length and has at most the max number of mismatches

Here is the caller graph for this function:

|  |  |  |  |
| --- | --- | --- | --- |
| static boolean umms.core.alignment.SmithWatermanAlignment.containsFullLengthUngappedMatch | ( | String | *seq1*, |
|  |  | String | *seq2*, |
|  |  | int | *seq1start*, |
|  |  | float | *matchScore*, |
|  |  | float | *mismatchScore*, |
|  |  | int | *maxMismatches* |
|  | ) |  |  |

static

Whether there is a full length ungapped match of sequence 2 to sequence 1, starting at specified position of sequence 1, allowing mismatches

Parameters
:   |  |  |
    | --- | --- |
    | seq1 | Sequence 1 |
    | seq2 | Sequence 2 |
    | seq1start | Required start position of match on sequence 1 |
    | matchScore | Match score |
    | mismatchScore | Mismatch score |
    | maxMismatches | Max allowable number of mismatches |

Returns
:   Whether the best ungapped alignment is full length and has at most the max number of mismatches

Here is the call graph for this function:

|  |  |  |  |
| --- | --- | --- | --- |
| static boolean umms.core.alignment.SmithWatermanAlignment.containsFullLengthUngappedMatch | ( | String | *seq1*, |
|  |  | String | *seq2*, |
|  |  | int | *maxMismatches* |
|  | ) |  |  |

static

Whether there is a full length ungapped match of the shorter sequence to the longer sequence, allowing mismatches Uses default match and mismatch scores

Parameters
:   |  |  |
    | --- | --- |
    | seq1 | Sequence 1 |
    | seq2 | Sequence 2 |
    | maxMismatches | Max allowable number of mismatches |

Returns
:   Whether the best ungapped alignment is full length and has at most the max number of mismatches

Here is the call graph for this function:

|  |  |  |  |
| --- | --- | --- | --- |
| static boolean umms.core.alignment.SmithWatermanAlignment.containsFullLengthUngappedMatch | ( | String | *seq1*, |
|  |  | String | *seq2*, |
|  |  | float | *matchScore*, |
|  |  | float | *mismatchScore*, |
|  |  | int | *maxMismatches* |
|  | ) |  |  |

static

Whether there is a full length ungapped match of the shorter sequence to the longer sequence, allowing mismatches

Parameters
:   |  |  |
    | --- | --- |
    | seq1 | Sequence 1 |
    | seq2 | Sequence 2 |
    | matchScore | Match score |
    | mismatchScore | Mismatch score |
    | maxMismatches | Max allowable number of mismatches |

Returns
:   Whether the best ungapped alignment is full length and has at most the max number of mismatches

Here is the call graph for this function:

|  |  |  |  |  |  |
| --- | --- | --- | --- | --- | --- |
| static String umms.core.alignment.SmithWatermanAlignment.getFullPrintableAlignment | ( | jaligner.Alignment | *alignment* | ) |  |

static

|  |  |  |  |
| --- | --- | --- | --- |
| static int umms.core.alignment.SmithWatermanAlignment.matchStartOnFirstSequence | ( | String | *seq1*, |
|  |  | String | *seq2*, |
|  |  | float | *matchScore*, |
|  |  | float | *mismatchScore*, |
|  |  | float | *gapOpenPenalty*, |
|  |  | float | *gapExtendPenalty*, |
|  |  | float | *minPctIdentity* |
|  | ) |  |  |

static

Get start position on first sequence of the Smith-Waterman alignment

Parameters
:   |  |  |
    | --- | --- |
    | seq1 | First sequence |
    | seq2 | Second sequence |
    | matchScore | Match score |
    | mismatchScore | Mismatch score |
    | gapOpenPenalty | Gap open penalty |
    | gapExtendPenalty | Gap extend penalty |
    | minPctIdentity | Min percent identity for alignment |

Returns
:   Start position of alignment on first sequence, or -1 if no suitable alignment exists

Here is the call graph for this function:

Here is the caller graph for this function:

|  |  |  |  |
| --- | --- | --- | --- |
| static int umms.core.alignment.SmithWatermanAlignment.ungappedMatchStartOnFirstSequence | ( | String | *seq1*, |
|  |  | String | *seq2*, |
|  |  | float | *matchScore*, |
|  |  | float | *mismatchScore*, |
|  |  | float | *minPctIdentity* |
|  | ) |  |  |

static

Get start position on first sequence of an ungapped Smith-Waterman alignment First sequence must be at least as long as second sequence

Parameters
:   |  |  |
    | --- | --- |
    | seq1 | Longer sequence |
    | seq2 | Shorter sequence |
    | matchScore | Match score |
    | mismatchScore | Mismatch score |
    | gapOpenPenalty | Gap open penalty |
    | gapExtendPenalty | Gap extend penalty |
    | minPctIdentity | Min percent identity for alignment |

Returns
:   Start position of alignment on first sequence, or -1 if no suitable alignment exists

Here is the call graph for this function:

## Member Data Documentation

float umms.core.alignment.SmithWatermanAlignment.DEFAULT\_GAP\_EXTEND\_PENALTY = 2

static

Default gap extend penalty for Smith Waterman

float umms.core.alignment.SmithWatermanAlignment.DEFAULT\_GAP\_OPEN\_PENALTY = 8

static

Default gap open penalty for Smith Waterman

float umms.core.alignment.SmithWatermanAlignment.DEFAULT\_MATCH\_SCORE = 5

static

Default match score for Smith Waterman

float umms.core.alignment.SmithWatermanAlignment.DEFAULT\_MISMATCH\_SCORE = -4

static

Default mismatch score for Smith Waterman

---

The documentation for this class was generated from the following file:

- java/umms/core/alignment/SmithWatermanAlignment.java


---

Generated on Fri Aug 15 2014 15:11:07 for ESAT by  

 1.8.7
